# Supplementary material for: Co-Expression Analysis of microRNAs and Proteins in Brain of Alzheimer’s Disease Patients
Source: Cells. 2022 Jan 4;11(1):163. doi: 10.3390/cells11010163 (PMC8750061; doi:10.3390/cells11010163)
Supplement: Supplementary file 1 [file cells-11-00163-s001.zip › cells-1505136-supplementary.pdf]

Co-expression analysis of microRNAs and proteins in brain of Alzheimer's Disease patients

Callum N. Watson,[1,†] Ghazala Begum,[1,†] Emma Ashman,[1] Daniella Thorn,[1] Kamal M. Yakoub,[1,2] Moustafa Al Hariri,[3] Ali Nehme,[1] Stefania Mondello,[4] Firas Kobeissy,[5] Antonio Belli,[1,2] Valentina Di Pietro [1,2,*]

[1] Institute of Inflammation and Ageing, University of Birmingham, Edgbaston, Birmingham B15 2TT, UK; cal97@live.co.uk (C.N.W.); ghazalabegum@hotmail.com (G.B.); eka894@alumni.bham.ac.uk (E.A.); DXT882@student.bham.ac.uk (D.T.); k.yakoub@bham.ac.uk (K.M.Y.); ali.hassan.nehme@gmail.com (A.N.); a.belli@bham.ac.uk (A.B.)

[2] Surgical Reconstruction and Microbiology Research Centre, National Institute for Health Research, Queen Elizabeth Hospital, Birmingham B15 2TH, UK

[3] Department of Emergency Medicine, American University of Beirut Medical Center, Beirut 1107 2020, Lebanon; ma147@aub.edu.lb

[4] Department of Biomedical and Dental Sciences and Morphofunctional Imaging, University of Messina, 98165 Messina, Italy; stm_mondello@hotmail.com

[5] Program for Neurotrauma, Neuroproteomics & Biomarkers Research, Departments of Emergency Medicine, Psychiatry, Neuroscience and Chemistry, University of Florida, Gainesville, FL 32611, USA; firasko@gmail.com

* Correspondence: v.dipietro@bham.ac.uk

† These authors contributed equally.

Table S1: DE-miRNAs of Braak stage III-IV compared to Braak stage V-VI and p-values ≤ 0.05 are reported.

MiRNAs	Braak III-IV *vs* Braak V-VI *p*-value
hsa-miR-185-3p	0.0079
hsa-miR-425-5p	0.0159
hsa-miR-425-3p	0.0079
hsa-miR-1539	0.0317
hsa-miR-212-3p	0.0317
hsa-let-7i-5p	0.0159
hsa-miR-185-5p	0.0159
hsa-miR-887-3p	0.0079
hsa-miR-503-5p	0.0317
hsa-miR-622	0.0317
hsa-miR-484	0.0317
hsa-miR-93-5p	0.0079
hsa-miR-877-5p	0.0159
hsa-miR-760	0.0159
hsa-miR-518a-3p	0.0159
hsa-miR-874-3p	0.0079
hsa-miR-98-5p	0.0079
hsa-let-7a-5p	0.0079
hsa-let-7e-5p	0.0159
hsa-let-7d-5p	0.0079

hsa-miR-106a-5p	0.0079
hsa-miR-181a-5p	0.0317
hsa-miR-125b-5p	0.0159
hsa-miR-331-3p	0.0317
hsa-miR-487b-3p	0.0317
hsa-miR-129-5p	0.0159
hsa-miR-130b-3p	0.0317
hsa-miR-296-5p	0.0159
hsa-miR-216a-5p	0.0317
hsa-miR-370-3p	0.0317
hsa-miR-330-5p	0.0159
hsa-miR-433-3p	0.0317
hsa-miR-1247-5p	0.0079
hsa-miR-550a-3p	0.0079
hsa-miR-489-3p	0.0317
hsa-miR-151a-5p	0.0317
hsa-miR-338-3p	0.0317

Table S2: DE-proteins of Braak stage III-IV compared to Braak stage V-VI and adj.*p*-Val are reported.

Protein	HGNC number	Braak III-IV *vs* Braak V-VI adj.*p*-Val
TNR11	TNFRSF11A	0.00055
COIA1 (endostatin)	COL18A1	$2.7x10^{-06}$
CTGF	CTGF	0.00018
SP1	SP1	0.0014
KI2L2	KIR2DL2	0.00028
MYBA	MYBL1	0.00012
IL34_MOUSE	Il34	0.00029
PGS2	DCN	0.0037
CSF3	CSF3	0.00022
I22R2	IL22RA2	0.045
POSTN	POSTN	0.0098
GLUC	GCG	0.015
BGH3	TGFBI	0.00029
SIGL9	SIGLEC9	0.0002
TSP1	THBS1	0.000056
A2AP	SERPINF2	0.0019
CCL28	CCL28	0.00023
JAK1	JAK1	0.017
CATB	CTSB	0.0039
LYAM2	SELE	0.0028
HLA-DR		$8.3x10^{-06}$

GNLY	GNLY	0.0028
CNTF	CNTF	0.000033
ESR1	ESR1	0.0002
TNF11	TNFSF11	0.029
PD1L2	PDCD1LG2	0.00012
IFNG	IFNG	0.00054
CXCL9	CXCL9	0.034
VGFR2	KDR	0.022
LAG3	LAG3	0.00018
CDCP1	CDCP1	0.00012
IGKC	IGKC	0.0012
ITA2B (CD41a)	ITGA2B	0.00023
EGLN	ENG	0.000084
NGAL	LCN2	0.000099
MK	MDK	0.041
THYG	TG	0.00023
KLK3	KLK3	0.00023
LEG9	LGALS9	0.00055
IL2RB	IL2RB	0.002
DAF	CD55	0.00015
ICAM1	ICAM1	0.017
NECT4	NECTIN4	0.00036
VTDB	GC	0.00018
CD72	CD72	0.00055
BCAM	BCAM	0.000084
CD44	CD44	0.033
P53	TP53	0.00055
CEAM5	CEACAM5	0.00055
SPA9	SERPINA9	0.00061
KLOTB	KLB	0.00055
MEP1A	MEP1A	0.00022
ULBP1	ULBP1	0.0011
CD22	CD22	0.0024
BLNK	BLNK	0.0079
CXL11	CXCL11	0.005
IL13_MOUSE	Il13	0.045
CAH9	CA9	0.00012
PTN1	PTPN1	0.00046
SLIP	NUGGC	0.0079
ULBP3	ULBP3	0.00055
CD52	CD52	0.015
IFNG	IFNG	0.015
TR11B	TNFRSF11B	0.0018
B2MG	B2M	0.018
CREB1	CREB1	0.0015
CR2	CR2	0.0013

TNFB	LTA	0.00089
EGLN	ENG	0.002
IGLC1	IGLC1	0.00065
HLA-ABC		$8.3x10^{-06}$
IL17C	IL17C	0.0042
FGF9	FGF9	0.0021
FPR1	FPR1	0.0046
UROK	PLAU	0.0019
AA2AR	ADORA2A	0.0021
TIE2	TEK	0.015
ITAE	ITGAE	0.017
IBP7	IGFBP7	$8.3x10^{-06}$
FABPL	FABP1	0.0021
FCG2B	FCGR2B	0.00064
IFNL3	IFNL3	0.0024
DAND5	DAND5	0.042
TNFL8	TNFSF8	0.045
IBP7	IGFBP7	0.00018
ITA4	ITGA4	0.011
WISP1	WISP1	0.0076
FINC	FN1	0.018
LYVE1	LYVE1	0.033
VGFR1	FLT1	0.017
KAIN	SERPINA4	0.028
CEAM1	CEACAM1	0.04
NRP1	NRP1	0.022
CCD50	CCDC50	0.000099
IL15	IL15	0.043
EGFR	EGFR	0.048
PARK7	PARK7	0.00055
GPI8	PIGK	0.045
UBE2T	UBE2T	0.02
BUB1	BUB1	0.0019
HGF	HGF	0.043
LIMD1	LIMD1	0.015
ANGL6	ANGPTL6	0.019
SAA1	SAA1	0.005
ANGL3	ANGPTL3	0.0073
ASTRA	GRAMD1A	0.015
AKT3	AKT3	0.004
HBEGF	HBEGF	0.00044
BIRC3	BIRC3	0.035
HPT	HP	0.005
CCR7	CCR7	0.00064
RA51C	RAD51C	0.0011
FGF23	FGF23	0.0013

BARD1	BARD1	0.033
PRIO	PRNP	0.00012
PRIO	PRNP	0.014
A1AT	SERPINA1	0.0084
RP9	RP9	0.03
CO1A1	COL1A1	0.0032
CFLAR	CFLAR	0.023
CRP	CRP	0.024

ELISA – protein validations

Methods

Protein preparation

Human FN1 (fibronectin) and human EGFR (Epidermal growth factor recptor) ELISA kits (Wuhan Fine Biotech, Wuhan, China) were used for the validation of Sciomics assay. Proteins were extracted using the instructions of manufacturer's ELISA kit. These were then frozen with a protease inhibitor before analysis using ELISA kits.

A BCA protein assay (Pierce, ThermoFisher, Waltham, MA, USA) was used to quantify the protein concentration after extraction with a standard curve. A linear regression line was used to make a standard curve to establish protein concentrations. Measurements were taken using a Tecan Infinite 200 Pro (Tecan, Männedorf, Switzerland).

An equal amount of protein was then added to the ELISA assay according the manufacturer's protocol.

Protein Analysis

An ELISA was carried out to quantify the FN1 and EGFR protein content within the samples.

A standard curve was used to identify the absorbance relevant for protein concentration. A sigmodial curve was used to interpolate the points and enable the protein concentration to be calculated. Due to the data being non-normally distributed based on a Shapiro-Wilk test. The Mann-Whitney test was used to assess the differences between groups (controls and Braak stage V-VI).

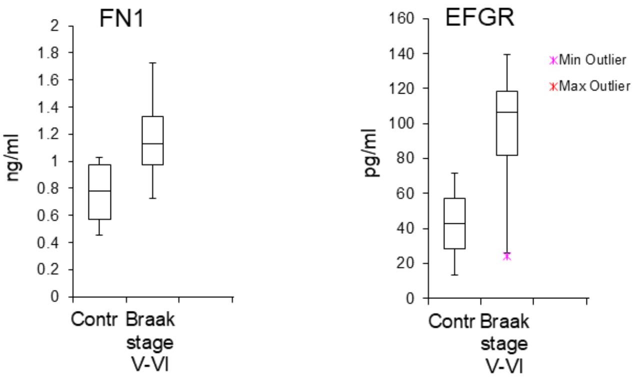

Figure S1: FN1 and EFGR protein concentrations after ELISA quantification

NO significant difference between control and Braak stage V-VI was found. However, there is an increase in the trend of both proteins when Braak stage V-VI samples are compared to controls, confirming and validating the Sciomic assay results.

Table S3: DE-miRNAs of AD brain tissues identifidied in this study were compared to DE-miRNAs of different biofluids identified in literature
(↓)= downregulation
(↑) = upregulation

miRNAs	Biofluids	Reference	Watson et al.
miR-146b-5p	Blood ↓	Wu et al. 2020 [1]	Brain ↓
miR132	Neural EVs ↓	Cha et al. 2019 [2]	Brain ↓
miR-212	Neural EVs ↓	Cha et al. 2019 [2]	Brain ↓
miR-425	PBMC ↑	Ren et al. 2016 [3]	Brain ↓
miR-106b	Serum ↓	Madadi et al. 2020 [4]	Brain ↓
miR-222	Serum ↓	Zeng et al. 2017 [5]	Brain ↓
miR-222	CSF ↑	Marchegiani et al. 2019 [6]	Brain ↓

miR-34c	Plasma ↑	Shi et al. 2020 [7]	Brain ↓
miR-34c	Serum ↑	Bhatnagar et al.2014 [8]	Brain ↓
miR-210-3p	Plasma ↑	Siedlecki-Wullich et al. 2019 [9]	Brain ↓
miR-545-3p	Plasma ↓	Cosin-Tomas et al. 2017 [10]	Brain ↓
Let-7i-5p	Serum ↓	Yuen et al. 2021 [11]	Brain ↓
miR-22-5p	Serum ↑	Yuen et al. 2021 [11]	Brain ↓
miR-210-3p	Plasam ↑	Yuen et al. 2021 [11]	Brain ↓
miR-106b-5p	Serum ↑	Yuen et al. 2021 [11]	Brain ↓
miR-212-3p	Blood ↓	Yuen et al. 2021 [11]	Brain ↓
miR-545-3p	Plasma ↓	Yuen et al. 2021 [11]	Brain ↓

Reference:

1) Wu; H. Z. Y.; Thalamuthu, A.; Cheng, L.; Fowler, C.; Masters, C. L.; Sachdev, P.; Mather, K.A.; the Australian Imaging Biomarkers and Lifestyle Flagship Study of Ageing. Differential blood miRNA expression in brain amyloid imaging-defined Alzheimer's disease and controls. *Alzheimers Res Ther.* **2020.** *12:59*. 10.1186/s13195-020-00627-0
2) Cha, D. J.; Mengel, D.; Mustapic, M.; Liu, W.; Selkoe, D. J.; Kapogiannis, D.; Galasko, D.; Rissman, R.A.; Bennett, D.A.; Walsh, D.M. miR-212 and miR-132 Are Downregulated In Neurally Derived Plasma Exosomes Of Alzheimer's patients. *Front. Neurosci.* **2019.** *13:1208*. 10.3389/fnins.2019.01208
3) Ren, R. J.; Zhang, Y. F.; Dammer, E. B.; Zhou, Y.; Wang, L.L.; Liu, X. H.; Feng, B.L.; Jiang, G.X.; Chen, S.D.; Wang, G.; Cheng, Q. Peripheral blood MicroRNA expression profiles in Alzheimer's disease: screening, validation, association with clinical phenotype and implications for molecular mechanism. *Mol Neurobiol.* **2016.** *53 5772–5781*. 10.1007/s12035-015-9484-8
4) Madadi, S.; Saidijam, M.; Yavari, B.; Soleimani, M. Downregulation of serum miR-106b: a potential biomarker for Alzheimer disease. *Arch Physiol Biochem.* **2020**. 10.1080/13813455.2020.1734842
5) Zeng, Q.; Zou, L.; Qian, L.; Zhou, F.; Nie, H.; Yu, S.; Jiang, J.; Zhuang, A.; Wang, C,; Zhang, H. Expression of microRNA222 in serum of patients with Alzheimer's disease. *Mol Med Rep.* **2019**. *16 5575–5579*. 10.3892/mmr.2017.7301
6) Marchegiani, F.; Matacchione, G.; Ramini, D.; Marcheselli, F.; Recchioni, R.; Casoli, T.; Mercuri, E.; Lazzarini, M.; Giorgetti, B.; Cameriere, V.; Paolini, S.; Paciaroni, L.; Rossi, T.; Galeazzi, R.; Lisa, R.; Bonfigli, A.; Procopio, A.D.; De Luca, M.; Pelliccioni, G.; Oliveri, F.Diagnostic

performance of new and classic CSF biomarkers in agerelated dementias. *Aging*. **2019**. 11, 2420–2429. doi: 10.18632/aging.101925

7) Shi, Z.; Zhang, K.; Zhou, H.; Jiang, L.; Xie, B.; Wang, R.; Xia, W.; Yin, Y.; Gao, Z.; Cui, D.; Zhang, R.; Xu, S. Increased miR-34c mediates synaptic deficits by targeting synaptotagmin 1 through ROS-JNK-p53 pathway in Alzheimer's disease. *Aging Cell.* **2020.** *19:e13125.* doi: 10.1111/acel.13125
8) Bhatnagar, S.; Chertkow, H.; Schipper, H. M.; Yuan, Z.; Shetty, V.; Jenkins, S.; Jones, T.; Wang, E. Increased microRNA-34c abundance in Alzheimer's disease circulating blood plasma. *Front Mol Neurosci.* **2019**. *7:2.* doi: 10.3389/fnmol.2014.00002
9) Siedlecki-Wullich, D., Catala-Solsona, J., Fabregas, C., Hernandez, I., Clarimon, J., Lleo, A.; Boada, M.; Saura, C.A.; Rodriguez-Alvarez, J.; Minano-Molina, A.J. Altered microRNAs related to synaptic function as potential plasma biomarkers for Alzheimer's disease. *Alzheimers Res Ther*. **2019.** 11:46. doi: 10.1186/s13195-019-0501-4
10) Cosin-Tomas, M., Antonell, A., Llado, A., Alcolea, D., Fortea, J., Ezquerra, M., Lleo, A.; Marti, M.J.; Pallas, M.; Sanchez-Valle, R.; Molimuevo, J.L.; Sanfeliu, C.; Kaliman, P. Plasma miR-34a-5p and miR-545-3p as early biomarkers of Alzheimer's disease: potential and limitations. *Mol Neurobiol.* ***2017.*** *54, 5550–5562*. doi: 10.1007/s12035-016-0088-8
11) Yuen, S.C.; Liang, X.; Zhu, H.; Jia, Y.; Leung, S.W. Prediction of differentially expressed microRNAs in blood as potential biomarkers for Alzheimer's disease by meta-analysis and adaptive boosting ensemble learning. *Alzheimers Res Ther.* 2021; 13(1):126. doi: 10.1186/s13195-021-00862-z.
